# Still Searching for a Suitable Molecular Test to Detect Hidden *Plasmodium* Infection: A Proposal for Blood Donor Screening in Brazil

**DOI:** 10.1371/journal.pone.0150391

**Published:** 2016-03-09

**Authors:** Giselle Fernandes Maciel de Castro Lima, Naomi W. Lucchi, Luciana Silva-Flannery, Alexandre Macedo- de- Oliveira, Angelica D Hristov, Juliana Inoue, Maria de Jesus Costa-Nascimento, Venkatachalam Udhayakumar, Silvia M Di Santi

**Affiliations:** 1 Departamento de Moléstias Infecciosas e Parasitárias, Faculdade de Medicina, Universidade de São Paulo, São Paulo, SP, Brasil; 2 Malaria Branch, Division of Parasitic Diseases and Malaria, Centers for Disease Control and Prevention, Atlanta, Georgia, United States of America; 3 Atlanta Research and Education Foundation, Decatur-GA, Atlanta, Georgia, United States of America; 4 Núcleo de Estudos em Malária, Superintendência de Controle de Endemias, Secretaria de Estado da Saúde de São Paulo /Instituto de Medicina Tropical de São Paulo, Universidade de São Paulo, São Paulo, SP, Brasil; Johns Hopkins Bloomberg School of Public Health, UNITED STATES

## Abstract

**Background:**

Efforts have been made to establish sensitive diagnostic tools for malaria screening in blood banks in order to detect malaria asymptomatic carriers. Microscopy, the malaria reference test in Brazil, is time consuming and its sensitivity depends on microscopist experience. Although molecular tools are available, some aspects need to be considered for large-scale screening: accuracy and robustness for detecting low parasitemia, affordability for application to large number of samples and flexibility to perform on individual or pooled samples.

**Methodology:**

In this retrospective study, we evaluated four molecular assays for detection of malaria parasites in a set of 56 samples previously evaluated by expert microscopy. In addition, we evaluated the effect of pooling samples on the sensitivity and specificity of the molecular assays. A well-characterized cultured sample with 1 parasite/μL was included in all the tests evaluated. DNA was extracted with QIAamp DNA Blood Mini Kit and eluted in 50 μL to concentrate the DNA. Pools were assembled with 10 samples each. Molecular protocols targeting 18S rRNA, included one qPCR genus specific (Lima-genus), one duplex qPCR genus/Pf (PET-genus, PET-Pf) and one duplex qPCR specie-specific (Rougemont: Roug-Pf/Pv and Roug-Pm/Po). Additionally a nested PCR protocol specie-specific was used (Snou-Pf, Snou-Pv, Snou-Pm and Snou-Po).

**Results:**

The limit of detection was 3.5 p/μL and 0.35p/μl for the PET-genus and Lima-genus assays, respectively. Considering the positive (n = 13) and negative (n = 39) unpooled individual samples according to microscopy, the sensitivity of the two genus qPCR assays was 76.9% (Lima-genus) and 72.7% (PET-genus). The Lima-genus and PET-genus showed both sensitivity of 86.7% in the pooled samples. The genus protocols yielded similar results (Kappa value of 1.000) in both individual and pooled samples.

**Conclusions:**

Efforts should be made to improve performance of molecular tests to enable the detection of low-density parasitemia if these tests are to be utilized for blood transfusion screening.

## Introduction

Malaria diagnosis remains a challenge for the detection of low parasitemias and more sensitive diagnostic tools are needed for the screening of asymptomatic infections in blood donations and transplantation products [1–3]. Microscopy remains the gold standard due to its low cost. However, it is time consuming, with a detection limit of 50–500 parasites per μL of blood, depending upon the microscopist’s experience [4,5]. Rapid diagnostic tests are easy to perform and require minimal specialized facilities or training; however, the currently available products cannot reliably detect parasitemia lower than 100 parasites/μL [4]. In contrast, nucleic acid amplification test (NAT), such as PCR, are able to detect the presence of low levels of DNA or RNA [6,7,8]. Malaria can be acquired through blood transfusion [9,10] due to asymptomatic donors with low parasite densities who are not screened for malaria before blood donation [11]. Many countries rely on travel history as an exclusion criterion for blood donation and have no approved test for blood screening. Nonetheless, the screening of blood before donation is an important malaria control effort that needs to be considered in order to limit the transmission of malaria via blood donation.

In Brazil, although the occurrence of malaria by blood transfusion is unknown, there are reports of this incidence, including lethal quartan malaria in an asplenic recipient. *Plasmodium malariae* was transmitted by a donor who reported displacement to an area where sporadic cases of asymptomatic autochthonous malaria are described. Microscopy was negative; however the PCR confirmed *P*. *malariae*. A second recipient from the same donor remained asymptomatic, with positive PCR [12]. Another case from the same region leading to transfusional transmission was reported [13] showing that clinical and epidemiological screening in blood banks may not be accurate for detection of asymptomatic carriers. The transmission in Brazil are predominantly in the Amazon Region where 99,6% of malaria cases occurs. *P*. *vivax* is the prevalent species with 84% of infections, followed by *P*. *falciparum* with 16% [14]. However, *P*. *malariae* is misdiagnosed with *P*. *vivax* and is reported mainly outside the Amazon Region [15]. Brazilian guidelines for hemotherapy services [16], recommends tests for *Plasmodium* detection in endemic areas and exclusion of donors with history of malaria in the 12 months preceding the donation or fever in the last 30 days. In non-endemic areas, candidates are excluded if coming from endemic areas in the last 30 days. After 30 days up to 12 months of displacement, testing for *Plasmodium* detection is required. In both endemic and non-endemic areas, donors who have had *P*. *malariae* infection are permanently ineligible. Although tests for *Plasmodium* detection are recommended, there is no indication of which test to use. Highly sensitive tests, such as molecular assays, would be required to detect malaria parasites in blood donors. Although many molecular tools are available for malaria parasite detection, deciding on the appropriate assay for large-scale screening of blood donors is not easy. The ideal assay needs to be the most accurate and robust for detecting low-density parasitemia, affordable for use in large numbers of samples and easy to perform. The pooling of samples is a commonly used practice, which has been used both for blood [17, 18, 8, 7] and for serum [19] samples. The pooling strategy is aimed at reducing both the cost and processing time of the assays and therefore it avails an ideal approach when the screening of large numbers of samples is required.

In this retrospective study, we evaluated four molecular assays for detection of malaria parasites in a set of samples previously evaluated by expert microscopy. In addition, we evaluated the effect of pooling samples on the sensitivity and specificity of the molecular assays.

## Materials and Methods

### Ethical Statements

This study was approved by the Research Ethics Committee at Medical School of the University of Sao Paulo, Brazil (protocol 205/12) and by the Scientific Committee of the Superintendence of Endemic Disease Control (SUCEN) (CC/229). Investigators from U.S. Centers for Disease Control and Prevention (CDC) were determined not to be engaged in human subjects research under this protocol (Reference #2013–390). Informed written consent forms were obtained from each study subject.

### Blood samples

**Fig 1:**
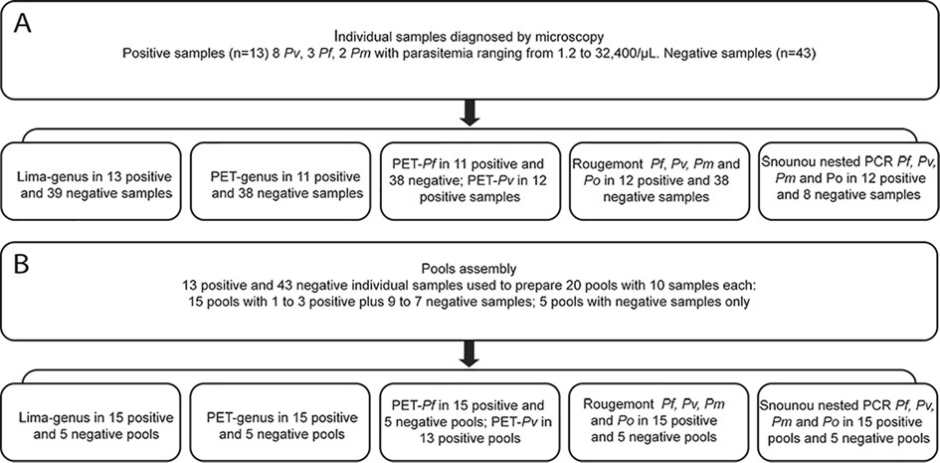
Blood samples and molecular assays used for detection of *Plasmodium*. (A) Individual samples detected by microscopy and number of samples assayed by different molecular tests. (B) Pooling of positive and negative samples assayed by different molecular tests.

Banked samples from 56 individuals [7] were used in this study. This included 13 *Plasmodium-*positive samples (4 of whom were asymptomatic) and 43 negative samples, the latter from individuals with no travel history to malaria endemic areas and no previous malaria infections (Fig 1). All samples were diagnosed by highly experienced microscopists, following the criteria recommended by the Brazilian Ministry of Health that determines the number of parasites/μL assuming a standard 6,000 leucocytes/μL [20]. A modification was made to the ministry of health’s recommendation in that 10,000 leukocytes, instead of 6,000 per slide were counted in order to quantify the number of parasites/μL. The parasitemia was therefore calculated as follows: (total parasites counted x 6,000)/10,000 leucocytes. Eight *P*. *vivax*, three *P*. *falciparum* and two *P*. *malariae* samples were identified with parasitemia ranging from 1.2 to 32,400/μL (Fig 1).

### DNA extraction and pool assembling

All positive and negative blood samples were processed in the same way. Blood was centrifuged to pellet the red blood cells (RBC). After plasma and buffy coat removal, the packed RBCs were lysed using 1% saponin and washed three times with distilled water. From this pellet, 200 μL was used for DNA extraction using the QIAamp DNA Blood Mini Kit (Qiagen®, Valencia, CA, USA). DNA was eluted using 50 μL of Elution Buffer to concentrate the DNA. An aliquot of packed RBCs from each positive and negative individual samples was used to prepare 20 pools of 10 samples each. To prepare pools, 300 μL of packed RBCs from each individual sample were combined into one tube. Each pool, now consisting of 3 mL of packed RBCs, was lysed and DNA extracted as previously described. Fifteen positive pools were prepared with one positive plus nine negative samples, two positive plus eight negative samples or three positive plus seven negative samples. Five pools were prepared with negative samples only (Fig 1). Each pool, now consisting of a 3 mL of packed RBCs, was lysed before being submitted to the DNA extraction procedure.

### Control samples and determination of limits of detection

Five dilutions of a *P*. *falciparum* culture at ring stage (kindly provided by Dr. MF Ferreira da Cruz, Fiocruz, RJ, Brazil), with parasitemia ranging from 350 to 0.035p/μL were used to determine the limits of detection of the various molecular tests. These controls were obtained by serial dilutions after calculating the number of parasites/μL: the number of parasites in 10,000 erythrocytes was counted in thin blood smear and calculated after counting the number of erythrocytes/μL using automatic counter. For the species-specific reactions, different *Plasmodium* species were used as positive controls: *P*. *falciparum* 3D7 (from CDC), *P*. *vivax*, *P*. *malariae* and *P*. *ovale* (from SUCEN) and a *P*. *falciparum* 1 parasite/μL (from SUCEN). Ultra-pure water and non-malaria samples were used as negative controls.

### Molecular assays

#### Lima genus quantitative real-time PCR (qPCR)

Lima qPCR for genus amplification (Lima-genus) was based on a previously published protocol [7] modified from Gama et al [21] to improve sensitivity and reduce costs. Briefly, the reaction was prepared using 2.5 μL of genomic DNA, 12.5 μL of 2x TaqMan® Universal PCR Master Mix, 500 nM of each genus-specific primer and 300 nM of the probe labeled with FAM™ and TAMRA™ (Applied Biosystems, Foster City, CA, USA). The threshold cycle (Ct) cut-off value of 37.28 was set based on the ROC curve as described before [7].

#### PET-PCR

PET-PCR was performed as described before [22], based on a self-quenching photo-induced electron transfer (PET) protocol using fluorogenic primers. The *Plasmodium spp* (PET-genus) and *P*. *falciparum* (PET-Pf) were performed in a duplex reaction. The 5’ end of the reverse primers was modified with the PET tag and labeled with FAM (for genus) and HEX (for *P*. *falciparum*). The protocol for *P*. *vivax* (PET-Pv) was performed in a separate reaction using HEX-labeled *P*. *vivax* primers. All amplifications were performed in a 20 μL reaction containing 2x TaqMan Environmental Master Mix 2.0 (Applied BioSystems, Foster City, CA, USA), 125 nM of each forward and reverse primer, except for the PET-Pf HEX primers which was used at 62.5 nM and 2 μL of DNA template. Any sample with a Ct value below 40.0 was considered positive.

#### Rougemont qPCR

Rougemont qPCR [23] was based on a multiplex protocol using a set of pan *Plasmodium* primers with four internal species-specific TaqMan®-probes for *P*. *falciparum* (Roug-Pf), *P*. *vivax* (Roug-Pv), *P*. *malariae* (Roug-Pm) and *P*. *ovale* (Roug-Po), performed in two simultaneous separate reactions. Four species-specific TaqMan®-probes were selected to differentiate between the species: *P*. *falciparum* labeled with FAM and BHQ1, *P*. *vivax* labeled with CY5 and BHQ2, *P*. *malariae* labeled with FAM and MGBNFQ and *P*. *ovale* labeled with HEX and MGBNFQ, designed to avoid interspecies polymorphic regions (Applied Biosystems, Foster City, CA, USA). The assay was performed in a final volume of 25 μL, containing 12.5 μL of 2x TaqMan Universal Master Mix (Applied Biosystems, Foster City, CA, USA), 200 nM of each primer, 80 nM of each probe and 2 μL of DNA template. The sample was considered positive by identifying the Ct within 40 cycles. Samples were assayed in duplicate in all the protocols. Results were considered indeterminate when the duplicates presented discordant results (one positive and one negative).

#### Snounou nested PCR

This protocol was performed according to Snounou et al [6] using genus-specific primers in the first PCR reaction and species-specific primers for *P*. *falciparum* (Snou-Pf), *P*. *vivax* (Snou-Pv), *P*. *malariae* (Snou-Pm) and *P*. *ovale* (Snou-Po) in the second PCR reaction. The fragments were separated by electrophoresis on a 1.5% agarose gel in Tris-borate-EDTA buffer and were visualized with ethidium bromide (Biorad, Hercules, CA, USA) or gel red through ultraviolet light.

### Statistical Analysis

A total of 56 individual samples and 20 pools were included in the analyses (Fig 1). Data were entered into Microsoft Excel 2007 and imported into EpiInfo, version 3.5.4 (Centers for Disease Control and Prevention, Atlanta, GA) for analysis. Kappa coefficients were used to quantify agreement between the two genus molecular tools using GraphPad. Confidence intervals were computed based on the exact binomial distribution. The sensitivities and specificities were calculated using microscopy as the reference standard.

## Results

### Limits of detection of the assays

Five dilutions of cultured *P*. *falciparum* with parasitemia ranging from 350 to 0.035p/μL were assayed using Lima-genus, PET-genus/PET-Pf and Snou-Pf protocols. The limit of detection for Lima-genus and Snou-Pf protocols was 0.35 parasite/μL with a Ct mean value of 35.55 for Lima-genus, whereas that for the PET-genus/PET-Pf was found to be 3.5 parasites/μL with a Ct mean value of 36.39 (Table 1). The detection limit of Rougemont protocol where published elsewhere and find to be similar to PET-Pf with detection of 3.2 parasites/μL [22].

**Table 1:** Limits of detection of molecular assays using standard controls obtained from *in vitro* culture.

*P*.*falciparum* standard controls	PET qPCR		Lima qPCR	Nested PCR
	PET-genus	PET-Pf	Lima-genus	Snou-Pf
350 p/μL	Pos	Pos	Pos	Pos
35 p/μL	Pos	Pos	Pos	Pos
3.5 p/μL	Pos	Pos	Pos	Pos
*1 p/μL	Neg	Indet	Pos	Pos
0.35 p/μL	Indet	Indet	Pos	Pos
0.035 p/μL	Neg	Neg	Indet	Neg

Pf = *P*. *falciparum*; Pos = Positive; Neg = Negative; Indet = indeterminate

* not from the same dilution series

### Comparative performance for individual unpooled samples

Thirty-nine of the 43 microscopy-negative samples were assayed individually by Lima-genus, 38 by PET-genus, PET-Pf and Rougemont qPCR and eight by Snounou nested PCR. The missing samples were due to lack of sufficient template DNA to perform all the assays. All microscopy negative samples tested were found to be negative by all molecular assays. Table 2 summarizes the results of all tests performed on the microscopy positive samples including the previous microscopy and parasitemia data. Thirteen samples were positive by microscopy: eight *P*. *vivax*, two *P*. *malariae* and three *P*. *falciparum*. The Lima-genus detected 10/13 positive samples including Sample 16, which was indeterminate (discordant replicates: one positive and one negative result). For blood screening, this sample was considered positive, as it would not be considered for blood donation. PET-genus assay detected 8/11 positive samples; two of the 13 positive samples were not tested due to lack of sufficient template DNA. The highlighted cells in Table 2 indicate discordant results among the tests evaluated including the species-specific tests. Samples 18 and 33, which were microscopy positive, were found to be negative by all molecular tests used. Sample 16 was found to be negative by all the tests except Lima-genus and Snou-Pm. From the eight samples detected as *P*. *vivax* by microscopy, four presented discordant results in the molecular tests: Sample 03 was negative by all *P*. *vivax* molecular tests, but positive for *P*. *falciparum* by Snou-Pf and for *P*. *ovale* by Snou-Po and Roug-Po. Sample 04 was positive for *P*. *malariae* by Snou-Pm and Roug-Pm.

**Table 2:** Comparison of molecular assays to microscopy in individual samples.

Sample Identification	Microscopy (parasite/ μL)	Lima-genus qPCR	PET-genus qPCR	Snou-Pf/Pv/Pm/Po nPCR	PET-Pf qPCR	PET-Pv qPCR	Rougemont qPCR
03	Pv (2,880/μL)	Pos	Pos	***Pf/Po***	Neg	**Neg**	**Po**
04	Pv (6,480/μL)	Pos	Pos	**Pm**	Neg	**Neg**	**Pm**
15	Pm (1.2/μL)	Pos	Pos	Pm	Neg	Neg	**Neg**
16	Pm (1.2/μL)	Pos*	**Neg**	Pm	Neg	Neg	**Neg**
18	Pv (2.4/μL)	**Neg**	**Neg**	**Neg**	**Neg**	**Neg**	**Neg**
21	Pf (4,800/μL)	Pos	Pos	Pf	Pf	Pos*	**Neg**
22	Pf (360/μL)	Pos	Pos	Pf	Pf	Neg	Pf
24	Pf (32,400/μL)	Pos		Pf		Neg	Pf
29	Pv (1,920/μL)	Pos	Pos	Pv	Neg	Pv	**Neg**
32	Pv (1.2/μL)	**Neg**		Pv		**Neg**	**Neg**
33	Pv 1.2/μL)	**Neg**	**Neg**	**Neg**	**Neg**		**Neg**
39	Pv (9.6/μL)	Pos	Pos	Pv	Neg	Pv	Pv
40	Pv (8,760/μL)	Pos	Pos	Pv	Neg	Pv	Pv

Empty boxes = Not Available; Pos* = indeterminate but called positive for blood screening; Pos = Positive; Neg = Negative; Pv = *P*. *vivax*; Pm = *P*. *malariae*; Pf = *P*. *falciparum*; Po = *P*. *ovale*. Discordant results with microscopy are in bold.

### Comparative performance for pooled samples

All 15 positive and five negative pools were tested with all molecular assays except for PET-Pv that was used for 13 of the 15 positive pools. Comparison of molecular assays in pooled samples is shown in Table 3. Pools with discordant results are highlighted. All negative pools were found to be negative by all molecular tests. Two pools (Pools 2 and 4) containing samples # 18 and 16, were negative by all molecular protocols. Pool 9 and 14 containing samples 04 and 03 were found to be Pm positive and Pf/Po respectively by the molecular tests although these were *P*. *vivax* samples by microscopy. Similar results were observed from the individual tests. Only one of the species was identified in the two pools with mixed infections (pools 5 and 10).

**Table 3:** Comparison of molecular assays in pooled samples.

Pool Identification	Positive Sample in the pool #/Microscopy Result	Lima-genus qPCR	PET-genus qPCR	Snou-Pf/Pv/Pm/Po nPCR	PET-Pf qPCR	PET-Pv qPCR	Rougemont qPCR
1	39/Pv	Pos	Pos	Pv	Neg	Pv	Pv
2	18/Pv	**Neg**	**Neg**	**Neg**	Neg		**Neg**
3	40/Pv	Pos	Pos	Pv	Neg	**Neg**	**Neg**
4	16/Pm	**Neg**	**Neg**	**Neg**	Neg		**Neg**
5	32/Pv;16/Pm;15/Pm	Pos	Pos	Pv	Neg	Neg	**Neg**
6	32/Pv	Pos	Pos	Pv	Neg	Pv	**Neg**
7	22/Pf	Pos	Pos	Pf	Pf	Neg	Pf
8	21/Pf	Pos	Pos	Pf	Pf	Neg	Pf
9	04/Pv	Pos	Pos	**Pm**	Neg	**Neg**	**Pm**
10	22/Pf;18/Pv	Pos	Pos	Pf	Pf	**Neg**	Pf
11	24/Pf	Pos	Pos	Pf	Pf	Neg	Pf
12	29/Pv	Pos	Pos	Pv	Neg	Pv	Pv
13	15/Pm	Pos	Pos	Pm	Neg	Neg	**Neg**
14	03/Pv	Pos	Pos	**Pf/Po**	**Neg**	**Neg**	**Po**
15	33/Pv;39/Pv	Pos	Pos	Pv	Neg	Pv	**Neg**
16	NPS	Neg	Neg	Neg	Neg		Neg
17	NPS	Neg	Neg	Neg	Neg		Neg
18	NPS	Neg	Neg	Neg	Neg		Neg
19	NPS	Neg	Neg	Neg	Neg		Neg
20	NPS	Neg	Neg	Neg	Neg		Neg

Empty boxes = Not Available; Pos = Positive; Neg = Negative; Pv = *P*. *vivax*; Pm = *P*. *malariae*; Pf = *P*. *falciparum*; Po = *P*. *ovale*; NPS = no positive sample. Discordant results with microscopy are in bold. Pools were assembled with 1 positive plus 9 negative, 2 positive plus 8 negative or 3 positive plus 7 negative samples.

The sensitivity and specificity was only calculated for the genus assays because the genus test provide a sufficient screening test for blood banks. Taking the expert microscopy used in this study as a reference test, the sensitivity in detecting parasites in individual samples was 76.9% in the Lima-genus assay and 72.7% in PET-genus assay. The specificity of the two genus assays was 100% (Table 4). Lima-genus and PET-genus showed a sensitivity of 86.7% in pooled samples.

**Table 4:** Sensitivity and specificity of the genus molecular assays in individual and pooled samples.

	**Individual Samples**	
	**Sensitivity % (95% CI)**	**Specificity % (95% CI)**
**Lima-genus**	76.9 (49.74–91.82)	100.0 (100.0–100.0)
**PET-genus**	72.7 (43.44–90.25)	100.0 (100.0–100.0)
	**Pooled Samples**	
	**Sensitivity % (95% CI)**	**Specificity % (95% CI)**
**Lima-genus**	86.7 (62.1–96.2)	100.0 (56.5–100.0)
**PET-genus**	86.7 (62.1–96.2)	100.0 (56.5–100.0)

Agreement (Kappa CI 95%) between the two genus molecular tests in individual and pooled samples was assessed using the Kappa agreement test. The individual assays showed a very good agreement with a K value of 0.930 (0.794–1.000) and for pooled assays a perfect agreement with K value of 1.000 (1.000–1.000).

## Discussion and Conclusions

In this retrospective study, we evaluated four molecular assays for detection of malaria parasites in a validated set of samples previously diagnosed by expert microscopy. In addition, we evaluated the effect of pooling samples on the sensitivity and specificity of the molecular assays. Considering the initial screening for detection of *Plasmodium* in blood banks, the use of a genus assay should be considered to decide whether to use blood for transfusion or not. For this purpose it is crucial high sensitivity of the assay, in order to avoid transfusional accident. The two genus protocols (Lima-genus and PET-genus) evaluated in this study yielded similar results although the Lima-genus demonstrated greater sensitivity than the PET-genus assay. This was in accordance with previous results, which showed the limits of detection of the Lima-genus and PET-genus tests to be 1p/μL and 3.5p/μL respectively [7, 22]. However, the adoption of microscopy as the gold standard should be considered carefully, since two positive samples resulted negative in all molecular tests, with no amplification in none of the duplicates. Our results indicate that the pooling strategy did not result in a significant loss of sensitivity implying that this strategy can be utilized to reduce costs and save time, as previously reported [7, 24].

Five of the 13-microscopy positive samples (15, 16, 18, 32, and 33) revealed interesting issues concerning malaria parasites diagnosis. Parasites in these samples were only detected after counting 10,000 leukocytes as opposed to the recommended 6,000, which greatly increased the probability of seeing the parasites present at low levels. For Samples 15, 16, 32 and 33, only two parasites were observed in 10,000 leukocytes and for Sample 18, four parasites were observed, giving a parasitemia of 1.2 and 2.4 parasites/μL, respectively. It is important to note that Sample 16 was from a blood donor that transmitted malaria to a recipient [13]. However, two of these samples (Samples 18 and 33), were not detected by any of the molecular tests evaluated. This raises the question as to whether the two samples (Samples 18 and 33) are truly positive samples or are false positive by microscopy. False positivity by microscopy has been previously documented [25] and unfortunately, this cannot be ruled out in our study. In addition, species misidentification by microscopy has previously been demonstrated [26]. In our study, two *P*. *vivax* samples by microscopy were shown to be *P*. *falciparum*/*P*. *ovale* (Sample 3) and *P*. *malariae* (Sample 4) by the three NAT tests. The occurrence of the *P*. *falciparum/P*. *ovale* case was surprising since *P*. *ovale* has not been reported in South America. Indeed, further investigation revealed that the P. *falciparum/P*. *ovale* case in our study was from an African immigrant. It has been demonstrated that molecular methods have better and more reliable performance when compared to commonly used microscopy [27].

The contribution of molecular-based protocols for blood bank screening resides in their ability to detect very low parasitemia. However, many inconsistencies have been shown by molecular tests when testing very low-parasite densities even when using the same protocol [27, 28]. Samples with very low-parasite densities assayed in duplicates occasionally showed amplification in only one aliquot, as observed in the standard controls diluted to 1 parasite/μL and 0.035 parasites/μL (Table 1) and in Sample 16 assayed by Lima-genus that had a Ct value of 37.4 (negative) in one well and 36.5 (positive) in the other well (Table 2). These observations were not entirely surprising because the reproducibility of PCR assays in the detection of samples with very low parasitemia was shown to alternate between positive and negative in about 38% of PCR replicates tested [28]. Similar inconsistencies, with low parasitemia samples, have been demonstrated in other studies [28, 29]. The exclusion for donation of blood with discordant results in the duplicate, considered here as indeterminate, helps to avoid the transfusion transmission of low parasite densities. Our results indicate that the nested PCR is a robust assay at detecting samples with low parasitemia. This is explained by the fact that nested PCR involves two rounds of DNA amplification leading to higher sensitivity. However, while this assay is more sensitive than the other molecular tests we evaluated, its applicability for large-scale screening of samples is limited since it is the most technically challenging of the four assays we evaluated.

The two genus molecular assays evaluated here (Lima-genus and PET-genus) may provide a reasonable alternative to microscopy for blood donor screening in Brazil if improvements in its sensitivity should be achieved. Although a sensitivity of 86.7% in pooled samples is not ideal for blood screening, efforts to improve the sensitivity is still required. High throughput methods using large volume of blood and concentrated DNA [1] associated with pooled sample processing using automation resources should be considered for screening of blood donors, reducing time and cost.

Although microscopy is the gold standard for malaria diagnosis, and where used in this study as the reference method, the NAT evaluated here exposed species misdiagnosis by microscopy. It is crucial discussing the use of microscopy as the gold standard in studies searching for sensitive techniques for the diagnosis of low parasitemia, since this technique has undergone no improvement since it was introduced. Misdiagnosis is a recurrent failure, particularly at low number of parasites. Additionally, despite thick blood smears is accepted as the universal gold standard, different methods for estimating parasitaemia are used in different laboratories, leading to difficulties in comparing data between studies [30].

Our study was limited by the small sample size and the fact that previously diagnosed samples were utilized. A large prospective study involving both symptomatic and asymptomatic cases will be required to help identify and evaluate a suitable molecular test to detect hidden *Plasmodium spp*. infection. Currently the search is still on for the best tool.
